# Heavy metal concentrations and inter-metal relationships in commercially available hair dyes of different colours: a chemometric evaluation

**DOI:** 10.1038/s41598-026-55782-5

**Published:** 2026-06-23

**Authors:** Omolara Jemimah Ojezele, Matthew Obaineh Ojezele

**Affiliations:** 1 Department of Chemistry/Biochemistry, Federal College of Animal Health and Production Technology, Moor Plantation, Ibadan, Nigeria; 2 Department of Pharmacology, Delta State University, Abraka, Delta State Nigeria

**Keywords:** Hair dyes, Heavy metals, Flame atomic absorption spectrometry, Chemometric analysis, Spearman correlation, Principal component analysis, Cosmetic safety, Chemistry, Environmental sciences

## Abstract

Cosmetic hair colouring products may contain trace inorganic contaminants introduced through pigments, raw materials, manufacturing processes, or packaging. Repeated use may contribute to low-level human and environmental exposure. This study determined the concentrations of seven heavy metals (Cr, Co, Cu, Pb, Cd, Ni, and Fe) in 21 commercially available hair dyes representing seven colour categories obtained from retail outlets in Ibadan, Nigeria. Samples were digested using a nitric acid–hydrogen peroxide mixture and analysed by flame atomic absorption spectrometry. Method reliability was verified using procedural blanks, replicate analysis, and spike recovery (95–104%). Metal concentrations (mg/kg) exhibited colour-dependent variability. Chromium ranged from 0.230 ± 0.245 mg/kg in green dyes to 2.53 ± 0.12 mg/kg in blue dyes, while copper showed the highest variability, reaching 7.03 ± 4.67 mg/kg in red dyes. Lead and nickel also varied across colour categories, with relatively higher nickel concentrations observed in blue dyes. Multivariate analyses (principal component analysis and hierarchical clustering) revealed partial grouping patterns and inter-metal associations, suggesting potential common sources linked to formulation components. Although concentrations were generally within available guidance ranges, the co-occurrence of multiple metals suggests the need for continued monitoring of cosmetic products as a screening-level indication of potential contamination concerns. Given the limited sample size and market scope, the findings should be interpreted as exploratory baseline data for cosmetic surveillance and environmental monitoring rather than definitive health risk characterization.

## Introduction

Cosmetic hair dyes are widely used consumer products across diverse populations for aesthetic, cultural, and personal identity purposes. Global demand for hair dye formulations has increased steadily, accompanied by expanding product diversity in colour, composition, and application type^1^. While safety evaluations of hair dyes have traditionally focused on organic colourants and sensitizing compounds, increasing attention has been directed toward the presence of heavy metals as unintended constituents of cosmetic formulations^2,3^.

From an analytical and environmental monitoring perspective, heavy metals in cosmetics are of concern due to their persistence, potential for bioaccumulation, and capacity to contribute to chronic low-level human exposure. Several heavy metals commonly detected in cosmetic products, including lead, cadmium, chromium, and nickel, have been associated with adverse biological effects following prolonged or repeated exposure, depending on exposure route and dose^4^. Metals such as chromium (Cr), nickel (Ni), cobalt (Co), lead (Pb), cadmium (Cd), copper (Cu), and iron (Fe) have frequently been reported in cosmetic products at trace concentrations^5,6^. Although cosmetics are not intended for ingestion, dermal contact and incidental environmental release during product use and disposal represent relevant exposure pathways^7,8^.

Regulatory control of heavy metals in cosmetic products varies globally. In the European Union, the intentional addition of heavy metals to cosmetics is prohibited, although trace impurities may be permitted under good manufacturing practices^9^. Other regulatory agencies, including the United States Food and Drug Administration and the ASEAN Cosmetic Committee, have established guidance values or impurity limits for selected heavy metals in cosmetic products to support product safety and quality control^10–12^. The absence of harmonised global limits highlights the importance of independent analytical monitoring studies to support evidence-based regulation and consumer protection.

Several studies have reported measurable concentrations of heavy metals in commercially available hair dyes across different markets. For example, Khalili et al.,^13^ reported detectable levels of chromium, nickel, and lead in hair dyes marketed in Iran, while Silva et al.,^14^ observed variable concentrations of several trace metals in hair dyes from Brazil and Paraguay. Similar investigations conducted in Nigeria have also identified the presence of heavy metals in cosmetic products, highlighting the importance of local surveillance studies^15^. These studies consistently reported the occurrence of trace metals such as chromium, nickel, lead, and copper in hair dye formulations, although the reported concentrations varied depending on factors such as pigment composition, raw material sources, and manufacturing processes. However, many of these investigations focus primarily on single-metal assessments or limited product categories and often lack multivariate evaluation of inter-metal relationships that could provide insight into shared contamination sources^5,16,17^.

Hair dye colour represents an important but underexplored factor influencing metal content. Different pigments and colour formulations may incorporate distinct inorganic compounds, metallic additives, stabilizers, or mineral-based ingredients, which may contribute to colour-dependent variation in metal concentrations^17^. From both analytical and regulatory perspectives, evaluating colour-dependent variability is important because consumers are exposed to diverse formulations that may contain distinct impurity profiles depending on dye composition and manufacturing processes. However, relatively few studies have systematically compared heavy metal concentrations across different colour categories of hair dye products. In addition, many existing investigations rely primarily on single-metal evaluations or basic descriptive statistics and rarely incorporate advanced chemometric approaches capable of revealing inter-metal relationships and potential shared contamination sources^18,19^). Studies integrating chemometric approaches with colour-based comparisons can therefore provide valuable baseline information for understanding variability patterns and supporting targeted cosmetic surveillance efforts.

The present study was designed to address these gaps by quantifying seven heavy metals (Cr, Co, Cu, Pb, Cd, Ni, and Fe) in commercially available hair dyes of different colours obtained from retail outlets in Ibadan, Nigeria. Using flame atomic absorption spectrometry supported by rigorous quality assurance procedures, this study investigates colour-specific variability in metal concentrations and examines inter-metal relationships through correlation analysis and multivariate techniques, including principal component analysis and hierarchical clustering.

The findings contribute regional baseline data on heavy metal occurrence in commercially available hair dyes and provide an integrated chemometric evaluation of inter-metal relationships and variability patterns across colour categories, supporting continued cosmetic surveillance and quality control efforts.

## Materials and methods

### Study area and sample collection

Commercially available hair dye products of different colours were purchased from retail cosmetic outlets within the metropolitan area of Ibadan, Oyo State, Nigeria. Ibadan is a major urban centre and an important commercial hub for cosmetic distribution, supplying products to surrounding regions and other parts of the country, making it suitable for consumer product surveillance studies. The sampled products were selected to represent commonly available commercial formulations encountered by consumers across different colour categories of hair dye products. Seven colour categories were selected for analysis: black, brown, red, green, white, purple, and blue. These colours represent commonly used hair dye formulations and incorporate a wide range of pigment compositions. For each colour category, three individual products representing different brands were randomly selected from available products at the point of purchase, resulting in a total of 21 samples. Although the inclusion of three products per colour category allowed preliminary comparison across colour groups, the relatively small sample size limits the inferential strength of statistical comparisons and supports interpretation of the findings primarily as exploratory baseline observations. All products were obtained in their original sealed packaging to minimize the risk of external contamination. Upon purchase, samples were labeled, stored in clean polyethylene containers, and transported to the laboratory for analysis.

### Sample preparation and acid digestion

Prior to digestion, each hair dye sample was thoroughly homogenized to ensure representative subsampling. Approximately 1.0 g of each homogenized sample was accurately weighed using an analytical balance and transferred into a clean digestion flask. Sample digestion was carried out using a wet acid digestion procedure commonly applied for cosmetic matrices^5,13^.

A mixture of 6 mL concentrated nitric acid (HNO₃, 65–70%, analytical grade) and 1 mL hydrogen peroxide (H₂O₂, 30%, analytical grade) was added to each flask. The digestion mixture was heated on a temperature-controlled hot plate at approximately 110–120 °C for about 30 min until a clear solution was obtained, indicating substantial decomposition of the sample matrix.

After cooling to room temperature, the digests were filtered through Whatman No. 42 filter paper to remove insoluble residues. The filtrates were quantitatively transferred into 50 mL volumetric flasks and diluted to volume with deionized water prior to instrumental analysis.

All glassware used throughout the procedure was pre-cleaned by soaking in 10% (v/v) nitric acid and rinsed thoroughly with deionized water to minimize the risk of external metal contamination.

### Instrumental analysis

Procedural blanks were analyzed alongside samples to monitor potential contamination and background signals during sample preparation and analysis. Blank readings for all analyzed metals were below the instrumental limits of detection. Each digested sample was analyzed in triplicate, and the mean concentration was reported. Results were expressed on a mass basis as milligrams per kilogram (mg/kg) of hair dye product.

### Quality assurance and quality control

Analytical precision was assessed through replicate measurements, with relative standard deviation (RSD) values generally below 5%, demonstrating good reproducibility of the method. Limits of detection (LOD), calculated as three times the standard deviation of procedural blanks, were sufficiently low to allow reliable quantification of all target metals. The calculated LOD values (mg/kg) were 0.01 for Cr, 0.005 for Co, 0.01 for Cu, 0.01 for Pb, 0.003 for Cd, 0.01 for Ni, and 0.05 for Fe. Values below the detection limit were not observed in the analyzed samples. Results are presented as mean ± standard deviation.

The spiked samples were subjected to the same digestion and analytical procedures as unspiked samples. Percentage recovery was calculated by comparing the measured concentration after spiking with the expected concentration using the expression:

$${\mathrm{Recovery}}{\mkern 1mu} \left( \% \right) = \left[ {\left( {{\mathrm{C}}_{{{\mathrm{(spiked)}}}} - {\mathrm{C}}_{{{\mathrm{(unspiked)}}}} } \right)/{\mathrm{C}}_{{{\mathrm{(added}})}} } \right] \times {\mathrm{1}}00$$

where C₍spiked₎ is the concentration measured in the spiked sample, C₍unspiked₎ is the concentration in the original sample, and C₍added₎ is the known concentration of the added standard. Recoveries ranged from 95 to 104%, indicating satisfactory digestion efficiency and analytical accuracy with minimal matrix interference.

As the study focused on commercially available products rather than controlled experimental samples, no external control samples were included. Instead, data reliability was ensured through the combined application of procedural blanks, calibration verification, replicate analysis, and recovery studies.

### Data analysis and statistical methods

All statistical analyses were performed using R statistical software (version 4.3.0) and IBM SPSS Statistics (version 25).

Descriptive statistics were used to summarize the concentrations of heavy metals across the different hair dye colour categories. One-way analysis of variance (ANOVA) was employed to assess statistically significant differences in metal concentrations among colours, with statistical significance defined at *p* < 0.05. Given the relatively small sample size within each colour category (*n* = 3), ANOVA was applied primarily as an exploratory statistical tool for detecting general patterns of variability rather than for definitive population-level inference. Where appropriate, pairwise comparisons between colour categories were performed using the Mann–Whitney *U* test to evaluate differences in metal concentrations between groups, particularly in cases where normality assumptions may not be satisfied. Because of the limited sample size, these pairwise comparisons should be interpreted cautiously as exploratory analyses with reduced statistical power.

Spearman rank correlation analysis was conducted to evaluate inter-metal relationships and potential co-occurrence patterns, as this non-parametric approach is more appropriate for datasets that may not meet the assumptions of normality.

Multivariate statistical analyses, including principal component analysis (PCA) and hierarchical cluster analysis (HCA), were applied to identify dominant patterns of variability and natural groupings within the dataset. PCA was used to reduce data dimensionality and visualize relationships between samples and metals, while HCA results were presented as a heatmap based on Spearman correlation coefficients. These multivariate analyses were applied as exploratory chemometric tools for pattern recognition and visualization of inter-metal associations within the dataset.

Contamination factor (CF) and pollution load index (PLI) were calculated as supplementary indices to evaluate the degree of metal contamination across samples.

CF values were calculated using the mean concentration of each metal within each colour category.

The contamination factor for each metal was calculated as:

$${\mathrm{CF}}\, = \,{\mathrm{C}}_{{{\mathrm{(sample)}}}} /{\mathrm{C}}_{{{\mathrm{(background)}}}}$$

where C₍sample₎ is the measured concentration of the metal in the hair dye sample (mg/kg), and C₍background₎ is the reference background concentration (mg/kg).

The pollution load index was calculated as the geometric mean of the contamination factors of all analyzed metals:

$${\mathrm{PLI}} = \left( {{\mathrm{CF}}_{{\mathrm{1}}} \times {\mathrm{CF}}_{{\mathrm{2}}} \times \ldots \times {\mathrm{CF}}_{{\mathrm{n}}} } \right)^{{1/{\mathrm{n}}}}$$

where CF₁, CF₂ … CFₙ are the contamination factors of individual metals, and *n* is the total number of metals analyzed.

For the purpose of calculating contamination factors, conservative reference background concentrations were adopted from a combination of available international cosmetic guidance values, including the U.S. Food and Drug Administration draft guidance and the ASEAN Cosmetic Directive^11,12^; ASEAN guidelines on cosmetic contaminant limits). These reference values were used solely as comparative benchmarks for environmental monitoring and do not represent legally enforceable limits. Accordingly, CF and PLI were applied as adapted comparative indices for evaluating relative contamination patterns and should not be interpreted as standard cosmetic health risk assessment tools.

For interpretation, CF values were classified as follows: CF < 1 (low contamination), 1 ≤ CF < 3 (moderate contamination), 3 ≤ CF < 6 (considerable contamination), and CF ≥ 6 (high contamination). PLI values were interpreted as follows: PLI < 1 indicates low overall contamination, PLI = 1 indicates baseline levels, and PLI > 1 indicates elevated contamination.

The application of contamination factor and pollution load index as comparative indices for assessing cumulative metal contamination is commonly employed in environmental monitoring studies of consumer products and environmental media^16,19^.

## Results and discussion

### Heavy metal concentrations in hair dyes

**Fig. 1:**
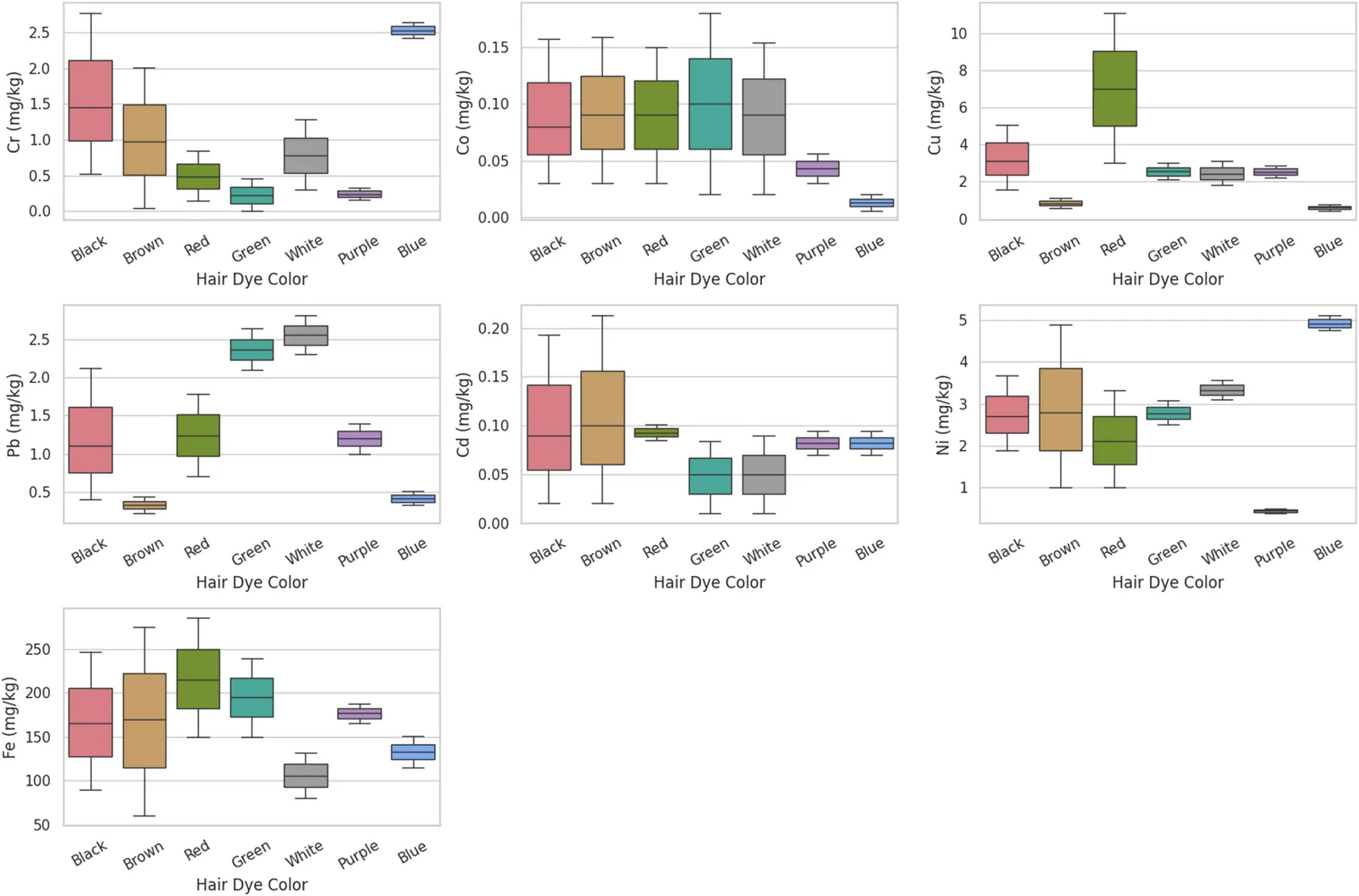
Multi-panel boxplot of heavy metals in hair dyes. Each boxplot represents the distribution of metal concentrations across three independent samples within each colour category (*n* = 3). The central line indicates the median, the box represents the interquartile range (IQR), and whiskers indicate the range of the data.

The concentrations of chromium (Cr), cobalt (Co), copper (Cu), lead (Pb), cadmium (Cd), nickel (Ni), and iron (Fe) measured in the analysed hair dye products are summarised in Table 1. While Table 1 presents the mean concentrations and associated variability for each colour category, the multi-panel boxplots provide a visual representation of the data distribution and highlight variability and potential outliers across colour groups (Fig. 1). Overall, detectable levels of all seven metals were observed across the different hair dye colours, although the concentrations varied across colour categories.

**Table 1:** Mean ± SD of heavy metals in different hair dye colours (mg/kg).

Metal	Black (B)	Brown (Br)	Red (*R*)	Green (G)	White (W)	Purple (*P*)	Blue (Bl)	*p*-value (ANOVA)
Cr	1.58 ± 1.13	1.01 ± 0.98	0.490 ± 0.350	0.230 ± 0.230	0.788 ± 0.505	0.243 ± 0.088	2.53 ± 0.11	0.004
Co	0.089 ± 0.064	0.093 ± 0.065	0.090 ± 0.060	0.100 ± 0.080	0.088 ± 0.067	0.043 ± 0.013	0.013 ± 0.007	0.477
Cu	3.26 ± 1.74	0.87 ± 0.284	7.03 ± 4.05	2.55 ± 0.45	2.44 ± 0.66	2.54 ± 0.34	0.61 ± 0.16	0.008
Pb	1.21 ± 0.86	0.33 ± 0.105	1.24 ± 0.54	2.37 ± 0.27	2.56 ± 0.26	1.20 ± 0.20	0.41 ± 0.09	< 0.001
Cd	0.101 ± 0.087	0.111 ± 0.097	0.093 ± 0.008	0.048 ± 0.037	0.050 ± 0.040	0.082 ± 0.012	0.082 ± 0.012	0.724
Ni	2.76 ± 0.88	2.90 ± 1.95	2.14 ± 1.17	2.79 ± 0.30	3.34 ± 0.24	0.448 ± 0.061	4.93 ± 0.18	0.002
Fe	167 ± 78	168 ± 107	217 ± 68	195 ± 45	106 ± 26	177 ± 12	133 ± 18	0.392

Values represent mean ± standard deviation of three commercially available brands within each colour category (*n* = 3). Individual brands were anonymized to avoid commercial bias. p-values represent one-way ANOVA comparing metal concentrations across colour categories; statistical significance was set at *p* < 0.05.

Metal concentrations exhibited pronounced colour-dependent variability (Fig. 1). Chromium concentrations were comparatively higher in blue dyes and lower in green dyes, which is consistent with possible differences in pigment composition or raw material inputs among colour formulations^5,17^. Cobalt concentrations remained relatively low across all colour categories, suggesting that cobalt may occur primarily as a trace impurity rather than as a deliberate formulation component in these products^16,14^.

Copper exhibited substantial variability among colour categories, with comparatively higher concentrations observed in red dyes and lower concentrations in blue dyes. This pattern is consistent with previous reports describing the use of copper-containing pigments in selected cosmetic colour formulations^17^. Iron was detected at comparatively higher concentrations than the other analysed metals across all colour categories, which may reflect its widespread occurrence in mineral pigments and filler materials commonly used in cosmetic products^2,3,20^.

Lead and nickel concentrations also varied across colour categories, with relatively elevated nickel concentrations observed in blue dyes. Cadmium concentrations remained comparatively low across all analysed samples; however, the consistent detection of cadmium across colour groups remains analytically relevant because trace metal impurities may accumulate through repeated cosmetic use. The observed occurrence of trace levels of heavy metals across all analysed hair dye samples is consistent with previous reports from different geographic regions, which have associated metal presence with pigments, raw materials, and manufacturing processes^5,17,14^.

The occurrence of trace metals observed in the present study is also consistent with previous Nigerian investigations reporting detectable levels of metals and metalloids in consumer cosmetic products. Yisa,^15^ documented the occurrence of metals and metalloids in cosmetic products sold in Abuja, Nigeria, supporting the importance of continued analytical surveillance and quality assessment of cosmetic products within the Nigerian market.

The measured concentrations were further compared with available international cosmetic impurity guidance values. Conservative limits suggested by the United States Food and Drug Administration and ASEAN cosmetic contaminant guidance documents indicate permissible impurity levels of approximately 10 mg/kg for lead, 3 mg/kg for cadmium, and 10 mg/kg for nickel in cosmetic products^10–12^. All measured concentrations of these metals in the analysed hair dye samples were below these guidance values, although variability across colour categories was observed.

Overall, these findings suggest that hair dye colour may influence heavy metal variability and may be associated with differences in pigment composition, raw material sourcing, and manufacturing inputs. To further evaluate whether the observed differences in metal concentrations across colour categories were statistically significant, inferential statistical analyses were performed.

### Differences in metal concentrations across colour categories

One-way analysis of variance (ANOVA) revealed statistically significant differences in the concentrations of chromium (Cr), copper (Cu), lead (Pb), and nickel (Ni) across hair dye colour categories (*p* < 0.05). In contrast, cobalt (Co), cadmium (Cd), and iron (Fe) did not show statistically significant differences (*p* > 0.05).

To further explore these differences, pairwise comparisons were performed using the Mann–Whitney *U* test. However, these analyses did not reveal statistically significant differences between most specific colour pairs (*p* > 0.05), despite the overall significance observed in the ANOVA results. Given the limited sample size within each colour category (*n* = 3), the pairwise analyses had reduced statistical power and were interpreted primarily as exploratory comparisons.

Observable trends were nevertheless noted, including relatively higher nickel concentrations in blue dyes and elevated lead concentrations in green and white dyes. These patterns were consistent with the descriptive statistics and overall ANOVA trends but were not sufficiently resolved to demonstrate statistically significant differences between individual colour pair combinations.

Overall, these findings suggest that colour-dependent differences in metal concentrations may exist at a global level across the analysed hair dye categories, while variability within groups and limited sample size constrain the statistical resolution of specific pairwise colour differences.

Detailed pairwise Mann–Whitney U test results are provided in Supplementary Table S1.

### Inter-metal relationships and correlation analysis

**Fig. 2:**
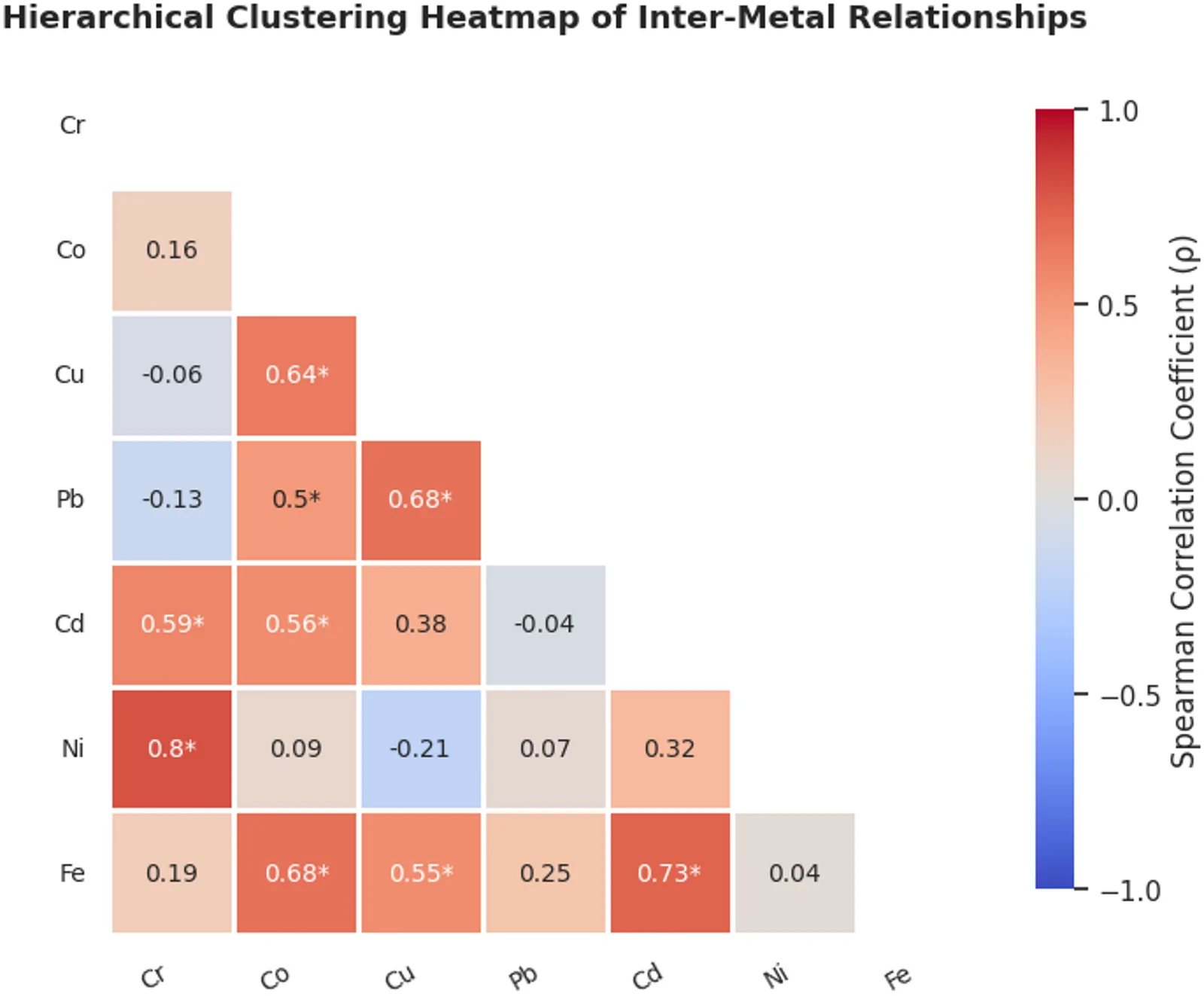
Hierarchical clustering heatmap of inter-metal relationships in hair dye samples based on Spearman’s rank correlation coefficients (ρ). The colour scale ranges from − 1 to + 1, where red indicates positive correlations and blue indicates negative correlations. Only the lower triangle of the matrix is shown for clarity. Asterisks (*) indicate statistically significant correlations (*p* < 0.05). Variables are ordered according to hierarchical clustering.

The hierarchical clustering heatmap (Fig. 2) was used to visualise inter-metal relationships based on Spearman’s rank correlation coefficients (ρ). The colour gradient represents the strength and direction of associations, with red indicating positive correlations and blue indicating negative correlations, while lighter colours represent weak or negligible relationships. The colour scale spans from − 1 to + 1. Statistical significance is denoted by asterisks (*), corresponding to *p* < 0.05. Because correlation coefficients may be sensitive to small sample sizes, the observed associations should be interpreted cautiously and primarily as exploratory indicators of potential inter-metal relationships.

Spearman correlation analysis revealed several statistically significant associations among the analysed metals. A strong positive correlation was observed between chromium (Cr) and nickel (Ni) (ρ = 0.80, *p* < 0.05), indicating a high degree of co-occurrence and suggesting possible shared formulation inputs, pigment composition, or raw material contributions. Copper (Cu) also showed a significant positive correlation with cobalt (Co) (ρ = 0.64, *p* < 0.05), while lead (Pb) exhibited significant positive associations with copper (ρ = 0.68, *p* < 0.05) and cobalt (ρ = 0.50, *p* < 0.05), which may reflect similarities in formulation components or manufacturing-related impurities.

Cadmium (Cd) demonstrated significant positive correlations with chromium (ρ = 0.59, *p* < 0.05) and cobalt (ρ = 0.56, *p* < 0.05), suggesting the possibility of related formulation inputs or contamination pathways. Iron (Fe) exhibited multiple significant positive associations, particularly with cadmium (ρ = 0.73, *p* < 0.05), cobalt (ρ = 0.68, *p* < 0.05), and copper (ρ = 0.55, *p* < 0.05), which is consistent with the potential contribution of iron-containing mineral components or additives to the co-occurrence of these metals in hair dye formulations.

In contrast, several negative or weak associations were observed. Chromium (Cr) showed a weak negative association with copper (Cu) (ρ = −0.06), while nickel (Ni) exhibited a weak negative association with copper (Cu) (ρ = −0.21). Chromium (Cr) also demonstrated a weak negative association with lead (Pb) (ρ = −0.13). Cadmium (Cd) exhibited a negligible negative association with lead (Pb) (ρ = −0.04). These weaker or inconsistent relationships suggest that some metals may be associated with distinct formulation components or manufacturing processes rather than possible shared inputs.

Overall, the observed correlation patterns suggest that multiple metals in hair dye products may be associated with possible shared formulation inputs, pigment composition, or raw material impurities, while others display more independent behaviour. The hierarchical clustering further supports these relationships by grouping metals with similar correlation profiles, providing exploratory insight into potential variability patterns and formulation characteristics.

### Multivariate analysis of heavy metal patterns (PCA and hierarchical clustering)

**Fig. 3:**
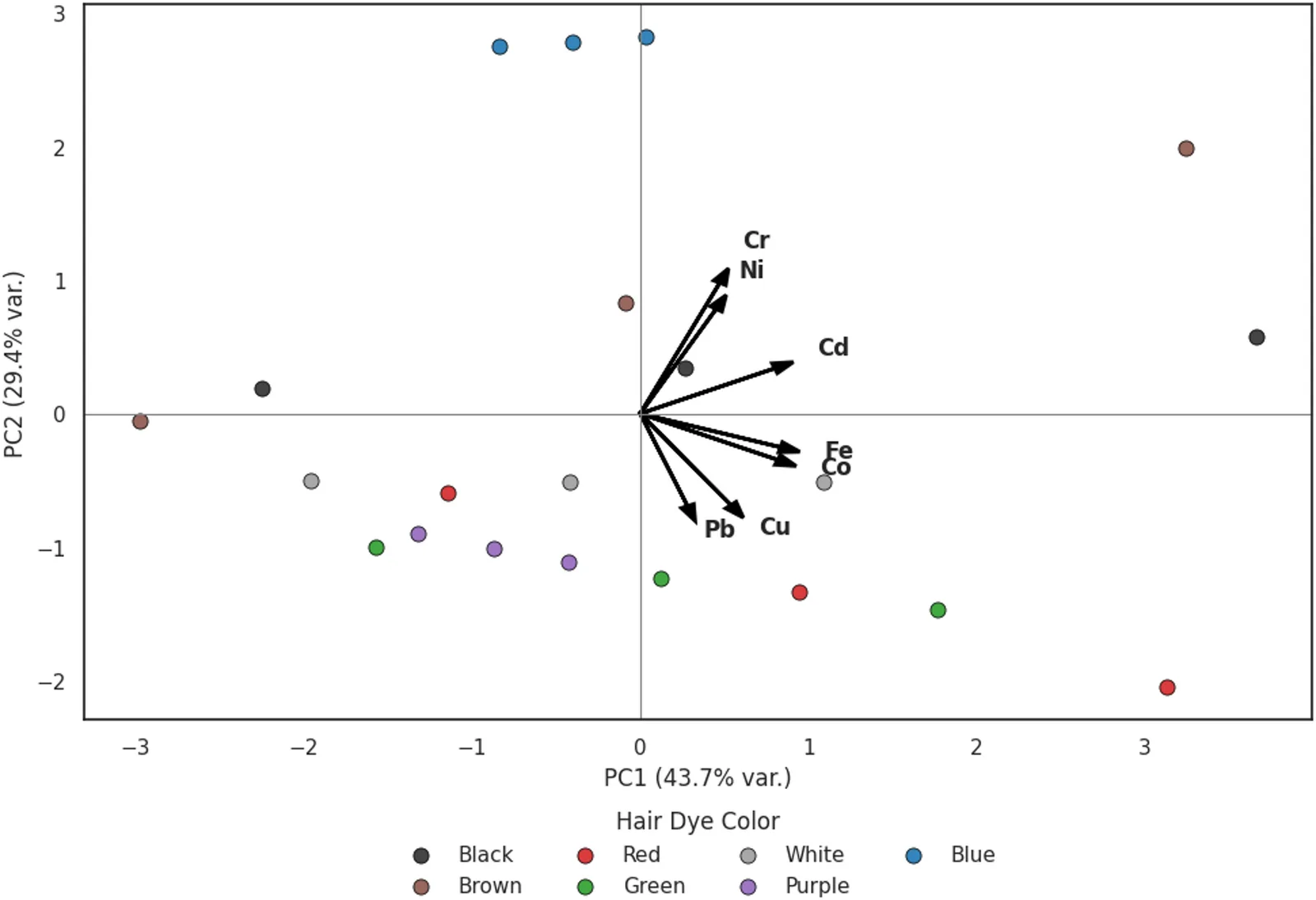
Principal component analysis (PCA) biplot showing relationships between heavy metals and hair dye samples of different colours. Points represent individual samples grouped by dye colour, while arrows represent loading vectors for Cr, Co, Cu, Pb, Cd, Ni, and Fe. The direction and length of each vector indicate the contribution and influence of the respective metal on the principal components. PC1 and PC2 explain 43.7% and 29.4% of the total variance, respectively.

Principal component analysis (PCA) was applied as an exploratory chemometric tool to evaluate the overall structure of the dataset and to identify dominant patterns governing heavy metal variability across the hair dye samples (Fig. 3). The first two principal components accounted for 73.1% of the total variance, with PC1 and PC2 explaining 43.7% and 29.4%, respectively. This relatively high cumulative variance suggests that the PCA captured major variability patterns within the dataset.

The PCA biplot (Fig. 3) revealed partial grouping patterns of samples according to hair dye colour categories, suggesting that formulation characteristics associated with colour may influence the overall metal composition. Chromium (Cr) and nickel (Ni) exhibited strong positive loadings along both PC1 and PC2, indicating that these metals are important contributors to the observed variability and tend to co-occur across samples. Cadmium (Cd) also showed a positive loading, primarily along PC1, reflecting its contribution to variation among certain dye categories.

In contrast, copper (Cu) and lead (Pb) were associated with negative loadings along PC2, suggesting an inverse relationship with metals positioned in the positive PC2 direction. Iron (Fe) and cobalt (Co) were closely aligned and exhibited similar loading directions, which is consistent with a related occurrence pattern and possible shared formulation inputs. These relationships are consistent with the positive correlations observed between Fe, Co, and Cd, as well as between Cr and Ni in Fig. 2.

Hierarchical clustering analysis (HCA) was similarly applied as an exploratory pattern-recognition approach to evaluate relationships among metals based on similarity patterns. Metals that exhibited strong positive associations in the correlation analysis, such as Cr and Ni, were positioned in similar regions of the PCA space, while metals with weaker or contrasting associations were more widely separated. This agreement between PCA, correlation analysis, and hierarchical clustering suggests consistency in the observed variability patterns across the applied multivariate methods.

Overall, the combined multivariate analyses suggest that the distribution of heavy metals in hair dye products may be influenced by possible shared formulation inputs, pigment composition, and raw material sources, while certain elements exhibit more independent variability. These findings are consistent with previous studies linking metal associations in cosmetic products to formulation components and manufacturing processes^18,16,19^.

### Contamination factor and pollution load index

In the absence of harmonised global regulatory limits for heavy metals in cosmetic products, conservative reference concentrations derived from available guidance documents, including the United States Food and Drug Administration (FDA) and ASEAN cosmetic contaminant guidance documents, were used as comparative benchmarks^10–12^. These reference concentrations were adopted solely to facilitate comparative evaluation of relative contamination intensity and should not be interpreted as definitive cosmetic safety thresholds or regulatory compliance standards.

The reference concentrations applied were 10 mg/kg for chromium (Cr), lead (Pb), and nickel (Ni); 3 mg/kg for cadmium (Cd); 50 mg/kg for copper (Cu); 5 mg/kg for cobalt (Co); and 500 mg/kg for iron (Fe). These comparative reference values were adapted from available FDA guidance documents for cosmetic impurities and supplementary contaminant guidance information provided by the ASEAN Cosmetic Committee, based on the closest corresponding available limits for cosmetic products and trace metal impurities^10–12^. These values were used to provide a consistent framework for comparative assessment rather than regulatory enforcement.

The contamination factor (CF) was calculated as the ratio of the measured metal concentration to the corresponding reference (background) concentration.

CF values can theoretically range from 0 (no detectable contamination) to values greater than 1, where values exceeding 1 indicate concentrations above the selected reference level. In environmental assessment practice, CF values are commonly interpreted as follows: CF < 1 indicates low contamination; 1–3 moderate contamination; 3–6 considerable contamination; and > 6 very high contamination. Although these interpretative categories are commonly applied in environmental monitoring studies, they are not specifically established for cosmetic risk assessment and were therefore used in this study only as comparative indicators of relative contamination intensity. In this study, CF values were calculated using the mean concentration of each metal within each colour category, rather than individual sample values, to provide a representative estimate for each dye group.

The pollution load index (PLI), calculated as the geometric mean of CF values across all analysed metals, provides an integrated measure of cumulative contamination relative to the selected reference concentrations. PLI values less than 1 indicate low overall contamination relative to reference concentrations, while values greater than 1 indicate elevated cumulative contamination. Accordingly, PLI values in this study were interpreted as comparative indicators of relative contamination burden rather than direct measures of cosmetic safety or health risk. The calculated CF and PLI values are presented in Table 2.

**Table 2:** Contamination factor (CF) and pollution load index (PLI) of heavy metals in hair dyes of different colours.

Colour	CF_Cr_	CF_Co_	CF_Cu_	CF_Pb_	CF_Cd_	CF_Ni_	CF_Fe_	PLI
Black	0.16	0.02	0.07	0.12	0.02	0.28	0.33	0.10
Brown	0.10	0.02	0.02	0.03	0.02	0.29	0.34	0.09
Red	0.05	0.02	0.14	0.12	0.02	0.21	0.43	0.11
Green	0.02	0.02	0.05	0.24	0.01	0.28	0.39	0.11
White	0.08	0.02	0.05	0.26	0.01	0.33	0.21	0.11
Purple	0.02	0.01	0.05	0.12	0.02	0.04	0.35	0.08
Blue	0.25	0.00	0.01	0.04	0.02	0.49	0.27	0.12
Reference concentration (mg/kg)	10	5	50	10	3	10	500	–

CF = C_(sample)_/C_(background)_; PLI = (CF_1_×CF_2_×…×CF_n_)^(1/n), where *n* is the number of analysed metals. Reference concentrations (mg/kg) used as comparative benchmark values were: Cr = 10, Co = 5, Cu = 50, Pb = 10, Cd = 3, Ni = 10, and Fe = 500. These reference concentrations were adapted from available FDA and ASEAN cosmetic guidance documents and were applied solely for comparative contamination assessment rather than regulatory compliance evaluation. CF values < 1 generally indicate relatively low contamination intensity compared with the selected reference concentrations, while values > 1 indicate concentrations above the comparative reference levels.

CF values for individual metals across all colour categories were generally less than 1, indicating low relative contamination intensity compared with the adopted reference concentrations. However, relatively higher CF values were observed for nickel, iron, lead, and chromium in specific colour groups. For example, nickel exhibited the highest CF in blue dyes (CF = 0.49), while lead showed comparatively higher CF values in green (CF = 0.24) and white dyes (CF = 0.26), reflecting colour-dependent formulation characteristics.

The PLI values ranged from 0.08 to 0.12 across the analysed hair dye colours, indicating relatively low cumulative contamination intensity relative to the selected reference concentrations. Although all PLI values remained below 1, the consistent occurrence of multiple metals across the analysed samples suggests the importance of continued cosmetic monitoring as a screening-level assessment of trace metal occurrence.

Combined exposure to multiple heavy metals has been discussed in toxicological literature because interactions among metals may influence biological responses under certain exposure conditions^4,21^. However, the present study did not include exposure modelling, dermal absorption assessment, or toxicological risk quantification; therefore, the CF and PLI results should be interpreted primarily as exploratory comparative indicators within the context of cosmetic surveillance and environmental monitoring.

## Conclusion

This study provides a systematic analytical evaluation of heavy metal concentrations in commercially available hair dyes of different colours obtained from retail outlets in Ibadan, Nigeria. Using flame atomic absorption spectrometry supported by rigorous quality assurance procedures, measurable levels of chromium, cobalt, copper, lead, cadmium, nickel, and iron were detected in all analysed products, with statistically significant colour-dependent variability.

Multivariate statistical analyses, including principal component analysis and hierarchical clustering, revealed inter-metal relationships and partial grouping patterns of samples according to colour category. These findings suggest that pigment composition, raw material sourcing, and manufacturing inputs may play important roles in shaping the metal profiles of hair dye formulations. The observed correlations between selected metals further suggest the possibility of related formulation inputs or manufacturing-associated impurities rather than isolated contamination events.

Contamination factor and pollution load index assessments, calculated using conservative combined FDA and ASEAN cosmetic guidance values as comparative reference concentrations, indicated generally low relative contamination intensity across all colour categories. Although PLI values remained less than 1, the consistent co-occurrence of multiple metals across samples supports the value of continued cosmetic monitoring and quality assessment efforts. While interactions among multiple heavy metals have been discussed in toxicological literature under certain exposure conditions^4^; Tchounwou et al., 2012), the present study did not include exposure modelling, dermal absorption assessment, or toxicological risk quantification. Therefore, the findings should be interpreted primarily as exploratory analytical data within the context of cosmetic surveillance and environmental monitoring.

This study is subject to certain limitations. The sample size was limited to products obtained from a single metropolitan market; however, this market represents a major commercial hub where cosmetic products are distributed wholesale to other parts of the state and across different regions of Nigeria. While this enhances the relevance of the findings, the results may not fully capture variability in products available in other settings.

The analysis focused on total metal concentrations without assessing metal speciation or dermal absorption potential. In addition, potential variability in user behaviour—such as differences in frequency of use, duration of application, and product handling practices across different hair dye types—was not considered and may influence actual exposure levels.

Overall, the results suggest that hair dye colour may influence heavy metal variability in cosmetic formulations. While the measured concentrations were generally within available international guidance ranges, the variability observed among colour categories supports the importance of continued quality control, routine surveillance, and baseline data generation for cosmetic products.

Future investigations incorporating broader geographic sampling, metal speciation analysis, dermal absorption modelling, and user behaviour assessment would provide deeper insight into variability patterns and potential exposure considerations associated with cosmetic use.

### Implications and future directions

The findings of this study provide useful insights into the occurrence and variability of heavy metals in commercially available hair dyes and highlight several areas for further investigation and potential regulatory consideration.

Routine surveillance of cosmetic products may be valuable for monitoring trace metal impurities and ensuring continued adherence to quality standards. In addition, improved raw material screening and manufacturing controls could help minimize the introduction of metal contaminants during production processes.

The observed colour-dependent variability suggests that certain formulation components may be associated with elevated metal concentrations, indicating a need for more targeted investigations into pigment composition and production inputs.

From a cosmetic surveillance and exposure assessment perspective, increased awareness of potential cumulative exposure to trace metals through repeated cosmetic use may be beneficial, particularly among frequent users.

Future research should focus on metal speciation, dermal absorption potential, and exposure modelling to better understand the bioavailability and health implications of these contaminants. Expanding studies to include a wider geographic distribution of products and additional cosmetic categories would further strengthen the evidence base for risk assessment and regulatory decision-making^20,21^.

## Data Availability

All data supporting the findings of this study are included within the article. Additional data related to this study are available from the corresponding author upon reasonable request.

## References

[1] de Souza, J. C. et al. B. Comprehensive review of hair dyes: Physicochemical aspects, classification, toxicity, detection, and treatment methods. *ACS Omega*. **10** (27), 28567–28586. 10.1021/acsomega.5c01576 (2025). 40686939 PMC12268732

[2] Abed, M. S., Moosa, A. A. & Alzuhairi, M. A. Heavy metals in cosmetics and tattoos: A review of historical background, health impact, and regulatory limits. *J. Hazard. Mater. Adv.* **13**, 100390. 10.1016/j.hazadv.2023.100390 (2024).

[3] Jităreanu, A., Toma, S., Sandu, I. & Vasilache, V. An overview of heavy metals in cosmetic products and their toxicological implications. *Appl. Sci.* **15** (24), 12883. 10.3390/app152412883 (2025).

[4] Balali-Mood, M., Naseri, K., Tahergorabi, Z., Khazdair, M. R. & Sadeghi, M. Toxic mechanisms of five heavy metals: Mercury, lead, chromium, cadmium, and arsenic. *Front. Pharmacol.* **12**, 643972. 10.3389/fphar.2021.643972 (2021). 33927623 PMC8078867

[5] Arshad, H., Mehmood, M. Z., Shah, M. H. & Abbasi, A. M. Evaluation of heavy metals in cosmetic products and their health risk assessment. *Saudi Pharm. J.* **28** (7), 779–790. 10.1016/j.jsps.2020.05.006 (2020). 32647479 PMC7335825

[6] Lara-Torres, S. et al. Determination and risk assessment of toxic metals in lipsticks from Europe and China. *J. Trace Elem. Med Biol.* **67**, 126792. 10.1016/j.jtemb.2021.126792 (2021). 34022566

[7] Khorshed, M. A., Ghuniem, M. M., Mahmoud, M. A. A. & Sorour, M. A. Multi-metric health risk assessment of heavy metal contamination in cosmetics: A comparative analysis of makeup, henna and skin creams. *Regul. Toxicol. Pharmacol.* **170**, 106128. 10.1016/j.yrtph.2026.106128 (2026). 42107517

[8] Akhtar, A., Kazi, T. G., Afridi, H. I. & Khan, M. Human exposure to toxic elements through facial cosmetic products: Dermal risk assessment. *Regul. Toxicol. Pharmacol.* **131**, 105145. 10.1016/j.yrtph.2022.105145 (2022). 35219764

[9] European Parliament and Council of the European Union. Regulation (EC) 1223/2009 on cosmetic products. *Official J. Eur. Union*. **L342**, 59–209 (2009). https://eur-lex.europa.eu/eli/reg/2009/1223/oj

[10] ASEAN Cosmetic Committee. *ASEAN Guidelines on Limits of Contaminants for Cosmetics, Release Version 3.0* (ASEAN Secretariat, 2023).

[11] Food, U. S. & Drug Administration (FDA). *Supporting document for recommended maximum lead level in cosmetic lip products and externally applied cosmetics* (FDA, Silver Spring, MD, 2016). https://www.fda.gov/cosmetics/cosmetics-guidance-documents/supporting-document-recommended-maximum-lead-level-cosmetic-lip-products-and-externally-applied

[12] Food, U. S., Administration, D. & FDA). *FDA’s testing of cosmetics for arsenic, cadmium, chromium, cobalt, lead, mercury, and nickel content* (FDA, Silver Spring, MD, 2022). https://www.fda.gov/cosmetics/potential-contaminants-cosmetics/fdas-testing-cosmetics-arsenic-cadmium-chromium-cobalt-lead-mercury-and-nickel-content

[13] Khalili, F., Mahvi, A. H., Nasseri, S., Yunesian, M. & Jahed, B. Health risk assessment of dermal exposure to heavy metals in chemical hair dyes. *Iran. J. Public. Health*. **48** (5), 903–913. 10.18502/ijph.v48i5.1807 (2019). PMC6717416 31523647

[14] Silva, G. M. et al. Investigation and health risk assessment of potentially toxic elements in hair-dye products sold in Brazil and Paraguay. *Sci* **7** (4), 160. (2025). 10.3390/sci7040160

[15] Yisa, A. G. et al. Health risk associated with metals and metalloids in frequently used oil perfumes sold at Karu market, FCT, Nigeria. *Discover Chem.* **2**, 186. 10.1007/s44371-025-00270-4 (2025).

[16] Kicińska, A. & Kowalczyk, M. Health risks from heavy metals in cosmetic products available in the online consumer market. *Sci. Rep.* **15**, 316. 10.1038/s41598-024-83477-2 (2025). 39747381 PMC11695611

[17] Mostafaii, G. et al. Determination of heavy metals in hair dye sold in the Iranian market: Dermal sensitivity and carcinogenicity assessment. *Biol. Trace Elem. Res.* **200** (3), 1464–1472. 10.1007/s12011-021-02738-7 (2022). 34033066

[18] Choi, S. et al. Concentrations and probabilistic health risks of seven metals in face and eye cosmetics across seven Asian countries. *Toxics* **14** (2), 167. 10.3390/toxics14020167 (2026). 41745841 PMC12944837

[19] Ahmed, M. et al. Multivariate statistical analysis of cosmetics due to potentially toxic metalloid contamination: Source identification for sustainability and human health risk assessment. *Sustainability* **16** (14), 6127. 10.3390/su16146127 (2024).

[20] Suliman, R. S. et al. Comparative analysis of the heavy metals content in selected colored cosmetic products at Saudi market. *J. Adv. Pharm. Tech. Res.* **12**, 430–434. 10.4103/japtr.JAPTR_150_21 (2021). PMC8588913 34820321

[21] Saah, S. A., Boadi, N. O., Sakyi, P. O. & Smith, E. Q. Human health risks of lead, cadmium, and other heavy metals in lipsticks. *Heliyon* **10** (23), e40576. 10.1016/j.heliyon.2024.e40576 (2024). 39654776 PMC11625256

